# Concentric Malar Lift in the Management of Lower Eyelid Rejuvenation or Retraction: A Clinical Retrospective Study on 342 Cases, 13 Years After the First Publication

**DOI:** 10.1007/s00266-018-1079-0

**Published:** 2018-02-20

**Authors:** Claude Le Louarn

**Affiliations:** 75116 Paris, France

**Keywords:** Mid-face lift, Blepharoplasty, Eyelid malposition, Vertical elevation, Skin excess

## Abstract

**Background:**

Lower eyelid rejuvenation can, unfortunately, induce scleral show even if the lower eyelid procedure is limited. This study was designed to assess the effectiveness and reliability of the concentric malar lift technique in two scenarios: the first, in rejuvenation of the mid-face and, the second, in reconstructive surgery for correction of congenital or acquired eyelids malposition.

**Methods:**

The concentric malar lift technique was first published by Le Louarn (Aesthet Plast Surg 28(6):359–372, 2004). This retrospective study was carried out by analyzing data on patients operated on between January 2010 and January 2016. Patients operated on before 2010 were excluded because barbed thread sutures were not used in the first version of the technique. Patients after January 2016 were excluded to ensure adequate follow-up, and so 342 patients are included in the study. A total of 256 cases (75%) were for aesthetic mid-face lifting, and 86 cases (25%) were reconstructive surgeries for lower eyelid retraction. A spacer graft was used in 30 of these reconstructive cases (35%). The mean follow-up time was 13.6 months. All the concentric malar lifting procedures included strengthening the lateral canthus, which is a key element of the procedure.

**Results:**

None of the patients developed secondary eyelid malposition, and all the cases of lower eyelid retraction displayed marked improvement both functionally and aesthetically. Two patients experienced loss of sensitivity of part in the infra-orbital nerve distribution for 4 months after the procedure.

**Conclusion:**

The concentric malar lift procedure enables the recruitment of a significant amount of skin into the lower eyelid: between 10 and 30 mm. It ensures better rejuvenation of the mid-face with minimal risk of lower eyelid malposition. In reconstruction of the lower eyelid lid, the concentric malar lift is able to reduce the need for skin grafting and a skin flap reducing the risks of visible scarring.

**Level of Evidence IV:**

This journal requires that authors assign a level of evidence to each article. For a full description of these Evidence-Based Medicine ratings, please refer to the Table of Contents or the online Instructions to Authors www.springer.com/00266.

## Introduction

Blepharoplasty is one of the most common cosmetic surgical procedures requested, yet malposition of the lower eyelid remains a frequent complication [2, 3]. In fact, even removing a small skin excess from the lower eyelid via a subciliary incision can induce an ectropion or scleral show.

**Fig. 1:**
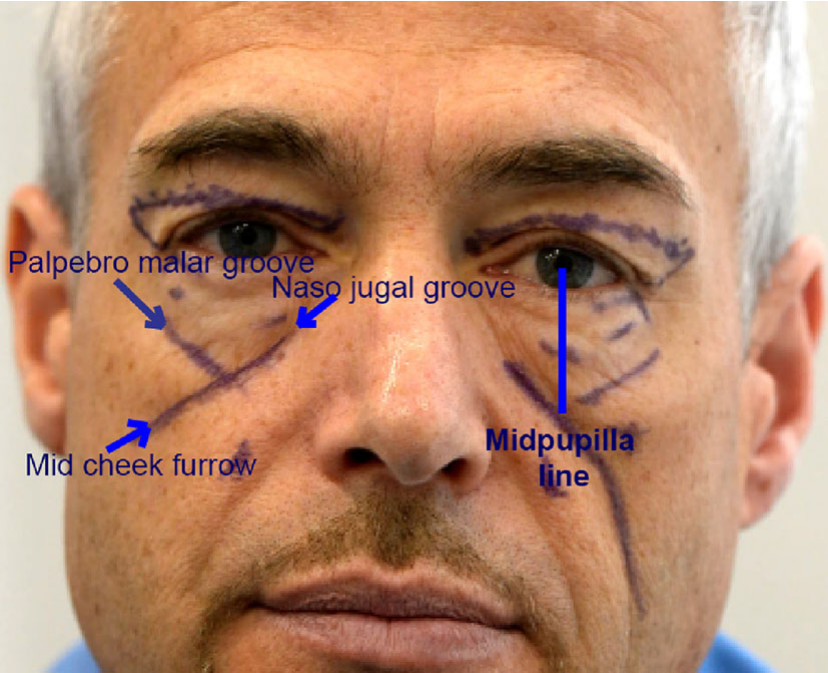
Above the malar mound, the palpebro-malar groove is marked, and below the mid-cheek furrow, the nasojugal groove is medial. These three lines are merging on the mid-pupilla line

To avoid this outcome, the orbicularis muscle is sometimes attached to the periosteum [4] at the lateral orbital rim and skin excess is removed at the level of the lateral canthus. Not only does this technique present a risk of secondary descent of the lateral canthus, but also it is not effective in lower eyelid rejuvenation, because this solution does not enable enough skin removal at the mid-pupillary, which is required to achieve effective lower eyelid rejuvenation. In fact, the junction between the 3 following lines, palpebro-malar groove, mid-cheek furrow and the nasojugal groove, is always situated on the mid-cheek in the vertical plane of the mid-pupilla line (Fig. 1). This junction represents the area of maximum movement and consequently of maximum skin excess at the lower eyelid level.

To correct lower eyelid malposition, a mid-face lift is sometimes performed. However, the risk of lower eyelid retraction is much more significant because the mid-face lift is a more extensive surgical procedure.

Obviously, a more extensive dissection induces a more extensive healing process and scarring, which increases the risk of tissue retraction. Tonnard and Verpaele [5] state that a ‘valuable and safe alternative to complicated, difficult, and potentially dangerous eyelid and mid-face rejuvenation techniques is micro-fat grafting.’

Micro-fat grafting is a safer technique, easy to perform and with acceptable results in many cases. However, micro-fat grafting cannot get rid of skin excess because so much fat would have to be injected that it would lead to ‘puffy eyes.’

When a mid-face lift is obviously necessary to obtain a satisfying result, the two main questions are:

1. How does the surgeon minimize the risk of secondary lower eyelid retraction?
2. How does the surgeon maximize the amount of skin to be removed safely in the mid-pupilla line? As previously explained, the more the skin that is removed via the subciliary incision in the mid-pupilla line, the better the outcome that should be obtained on the lower eyelid and mid-cheek.

In the author’s experience, the concentric malar lift is the only technique, which fulfills these two aims.

If we consider the causes of lower eyelid retraction, it can result from an isolated defect of one or all of the three lamellae.

The most common cause is an anterior lamella retraction due to excess skin excision, sometimes combined with an excess of orbicularis oculi muscle excision. When it is the intermediate lamella that is damaged, it results from a surgical trauma to the orbital septum followed by excessive scaring. Lastly when the posterior lamella is retracted, it nearly always results from surgical trauma of the conjunctiva and, unfortunately, it is frequently combined with trauma to the capsulopalpebral fascia and the retractor muscle. To be effective, surgical treatment must be adapted to the original damage process [2, 3].

The treatment of posterior lamella retraction requires a spacer graft, performed after horizontal incision of the mucosa and eventually of the capsulopalpebral fascia. A dermal fascia graft can be used for small defects, but a paramedian palatine mucosa graft, which is more rigid, is the ‘gold standard.’

The treatment of septal retraction (intermediate lamella damage) requires excision of the retracted area and use of a thin graft of temporal aponeurosis.

The treatment of anterior lamella retraction traditionally requires the use of a full-thickness skin graft or local flaps to replace the lost skin. But the proven efficacy and reliability of the concentric malar lift on anterior lamella retraction allow it to address the problem in most cases [6] without visible scars. Of course, the concentric malar lift is a more complicated procedure, much more demanding for the surgeon, and the recovery time is increased. Nevertheless, the technique is very safe, can recruit a lot of skin (1–3 cm) and can be performed through a subciliary incision alone. Moreover, the concentric malar lift rejuvenates the mid-face naturally because it enables relocation of the displaced mid-face volume upward into the desired position.

The aim of this publication is to share the author’s experience with the concentric malar lift, which achieves an effective and reliable mid-face lift, even in the 20% of cases in whom the postsurgical healing process has caused retraction. It is important to bear in mind that, no matter which mid-face procedure is used [7], retraction will occur in 20% of cases. The efficacy and reliability of the concentric malar lift technique has been effective even in cases presenting with major retraction of the anterior lamella due to previous surgery. Finally, even in reconstructive cases with eyelid malposition, postoperative retraction did not reduce the end result.

## Patients and Methods

This retrospective study includes patients operated on by the author between January 2010 and January 2016, 342 patients. Patients were selected from the author’s database, which includes patient demographic data, clinical pictures, pre-, per and postoperative analysis and complications. Women account for 232 patients, and men, 110 cases. Patient ages ranged from 21 to 89 years, with a mean age of 55.3 years.

When eyelid retraction was present before surgery, the degree of retraction was estimated in millimeters between the lower limbus and the palpebral margin during horizontal gaze and it was classified as mild (< 1 mm), moderate (2–3 mm) or severe (> 3 mm) [8].

The follow-up period was 8–24 months, with a mean of 13.4 months. Standard clinical pictures were taken before and after surgery, looking straightforward at rest, without contraction of the orbicularis oculi muscle and without using a flash to avoid hiding orbital relief and wrinkles. Per-operative photographs showed the height of skin removed and allowed the objective assessment of improvement in eyelid elevation in cases of eyelid retraction or in skin tension without change in position of the eyelid margin in aesthetic cases.

Improvement of ocular discomfort in reconstructive cases presenting with lower lid retraction, involved analysis of patient questionnaires completed preoperatively and at 1 and 8 months postsurgery (Table 1).

**Table 1:** Ocular discomfort evaluation on 86 cases presenting lower eyelid positioned between 1 mm and more than 3 mm from the limbus, congenitally or acquired

Symptoms	Preoperative cases—%	1 month after cases—%	8 months after cases—%
Pain	70 (81%)	35 (40%)	2 (2%)
Dryness	68 (79%)	12 (14%)	1 (1%)
Excessive tearing	29 (33%)	39 (45%)	0 (0%)
Tiredness	80 (93%)	60 (70%)	1 (1%)

Lower eyelid repositioning following a concentric malar lift is effective.

### Preoperative Evaluation

It is mandatory to perform a snap test to determine the palpebral tone before planning surgery to minimize scleral show.

In mid-face rejuvenation, a precise analysis of the position and function of both the lower eyelid and the lateral canthus is mandatory to determine the potential for aesthetic improvement.

In cosmetic modifications of eye shape, the concentric malar lift is the best option in the author’s opinion. Of course, the extent of modification must be carefully estimated in a tailor-made procedure.

Frequently, preoperative examination of the patient shows a deflated mid-face and fat grafting may be appropriate especially if patients request increased volume but must be assessed after the preexisting volume has been redistributed. A preoperative test with a picture of the patient lying down can show a sufficient volume in the mid-face.

In reconstructive treatment of eyelid retraction, patient examination and functional analysis need to be more detailed to determine precisely which lamella is predominantly involved in the pathogeneses of the eyelid retraction [5]. For this purpose, a vertical traction test pushing the lower eyelid up over the cornea with the surgeon’s finger is performed. Normally, the finger placed on the eyelid margin should easily reach the superior orbital rim. If the cause of eyelid retraction is the anterior lamella, a certain degree of resistance is perceived during elevation and recruiting skin from mid-face to lower eyelid eliminates this resistance. This indicates the potential efficacy of a mid-face lift to treat the retraction [6].

If recruiting skin from the mid-face does not produce any elevation and the excess skin slides over the ciliary margin, this indicates the retraction is posterior or, more rarely, medial. The surgical history will give additional information: if a conjunctival approach had been performed, the posterior lamella is, a priori, suffering from retraction, whereas if a septal reset or resection had been performed, the medial lamella is most likely to have retracted.

Finally, if the lateral canthus is too low or/and too medial, the association of a canthopexy is usually mandatory for aesthetic and/or functional reasons [7]. But canthopexy alone is not an alternative to a concentric malar lift because it will never be stable enough to definitively elevate the eyelid. Moreover, the tension induced by the canthopexy could give an unnatural ‘cat-eye’ appearance. The logical and safe solution is to elevate the mid-pupillary level with the mid-face lift and the canthal position with the canthopexy. When a canthopexy is associated with a concentric malar lift, the result is stable, because the canthopexy does not primarily tighten the lower eyelid [8].

During the procedure, it may be necessary to perform a buccal incision to locate the infra-orbital nerve. It is important to ensure dental health and hygiene are attended to before the procedure.

### Surgical Technique

The author first published about the mid-face lift in 1989 [9, 10]. The original technique for mid-face lifting was expanded from a preperiosteal oblique flap in 1989, through a subperiosteal oblique technique published in 1992 and 1994 [11, 12], to reach the concentric malar lift published in 2004 [1], 2006 [13] and 2007 [14].

At that time, the concentric malar lift was the only vertical subperiosteal mid-face lift described able to remove more than 15 mm of skin on the mid-pupilla line. It has to be noted that Hamra [15] published, in 1998, the first vertical approach to mid-face rejuvenation, but his dissection was preperiosteal and the lower eyelid vertical vector was created by a muscular flap at the lateral canthus level with limited efficacy on the mid-pupilla line rejuvenation.

In its first description (2004 [1]), the key points of the concentric malar lift technique were stated as:

- subciliary lower eyelid incision and one on the lateral part of the upper-eyelid,
- a 2-cm incision is made through the orbicularis at the lateral canthus level to perform a complete subperiosteal malar release,
- holes are drilled through the lateral and inferior orbital rim
- the elevation of the malar volume obtained with the suspension is concentric with the orbit,
- lateral canthopexy and lateral orbicularis oculi muscle suspension are performed

With the concentric malar lift the secondary retraction risk is controlled in 3 ways [16, 17]:

1. Limited orbicularis oculi muscle opening at the lateral canthus. Limited dissection should reduce the risk of retraction, whereas complete opening of the orbicularis oculi muscle used in some mid-face techniques creates a direct connection between the eyelid margin and the anterior malar area. This extent of dissection will result in descent of the lower eyelid if scarring produces retraction.
2. Fixation of the lateral canthopexy avoids descent of the posterior lamella
3. Suspension of the lateral orbicularis oculi muscle avoids descent of the anterior lamella

Since 2010, in all the 342 cases reported here, the concentric malar lift has been performed as described but with the addition of 2 barbed sutures used to elevate concentrically the lower and upper part of the malar volume, like a hammock, enabling creation of a double concentric elevation.

### Anesthesia

General anesthesia with local anesthesia injections was performed in all 342 cases. In the author’s practice, concentric malar lift with or without associated upper-eyelid surgery is always a day-case procedure. When a face lift is added, a one-night stay is required.

### Preoperative Markings and Infiltration

The curved lines of the palpebro-malar groove and of the nasojugal groove are marked in conjunction with the mid-cheek furrow (Fig. 1). The malar mound, which is important to ‘flatten’ surgically, is between the palpebro-malar groove and the mid-cheek furrow.

Infiltration is performed with epinephrine (0.5 mg per 300 ml saline), with ropivacaine 7.5 mg in 20 ml and lidocaine 20 mg/ml with epinephrine 0.005 mg/ml for 40 ml. It is injected superficially for the subcutaneous palpebral dissection and at the level of the periosteum for the subperiosteal release.

### Subcutaneous Dissection

The subciliary eyelid incision extends from the lacrymal point to 4 mm outside the lateral canthus.

The height of the subcutaneous dissection is the same as the height of skin to be resected in each case.

**Fig. 2:**
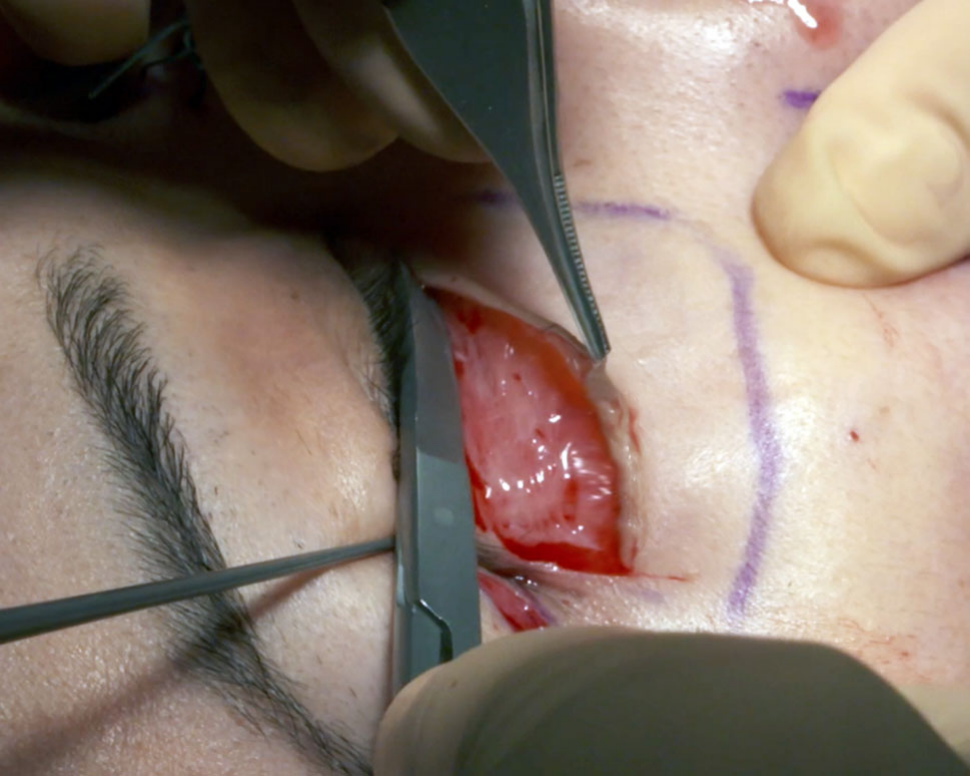
On that primary case of lower eyelid surgery, half the distance between the lid margin and the palpebro-malar groove, on the mid-pupilla line, is dissected

**Fig. 3:**
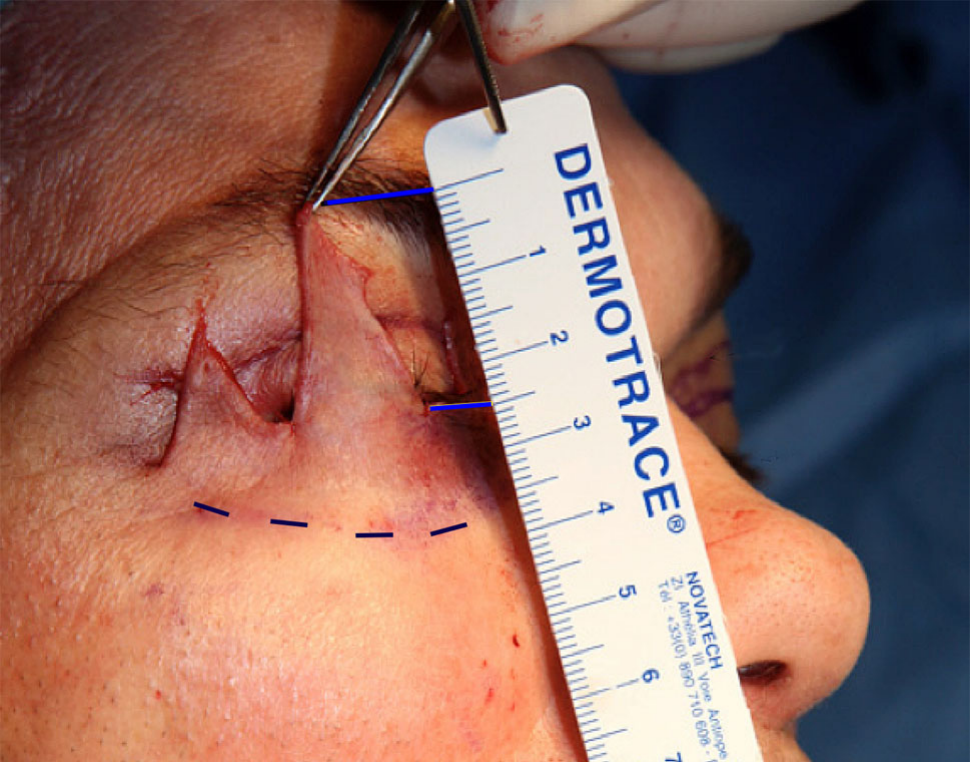
The height of tensed skin resected (25 mm) is half the distance between eye margin and palpebro-malar groove

A patient, who has not had previous lower eyelid surgery, will have half the distance between the eyelid margin and the mid-cheek junction to dissect and to resect (Fig. 2), usually between 10 and 25 mm of tensed skin excised (Fig. 3).

A patient who has previously undergone a standard lower eyelid blepharoplasty, with no retraction, will usually have between 5 and 15 mm removed. In this case the moderate skin excision is partially due to the 2 mm of skin already excised and mainly to the fibrotic, non-elastic, lower eyelid due to the healing of the fat bag resection.

Whereas a patient presenting with lower eyelid retraction due to previous surgery will have between 0 and 10 mm of skin excess.

### Upper-Eyelid Incision: Subperiosteal Dissection

**Fig. 4:**
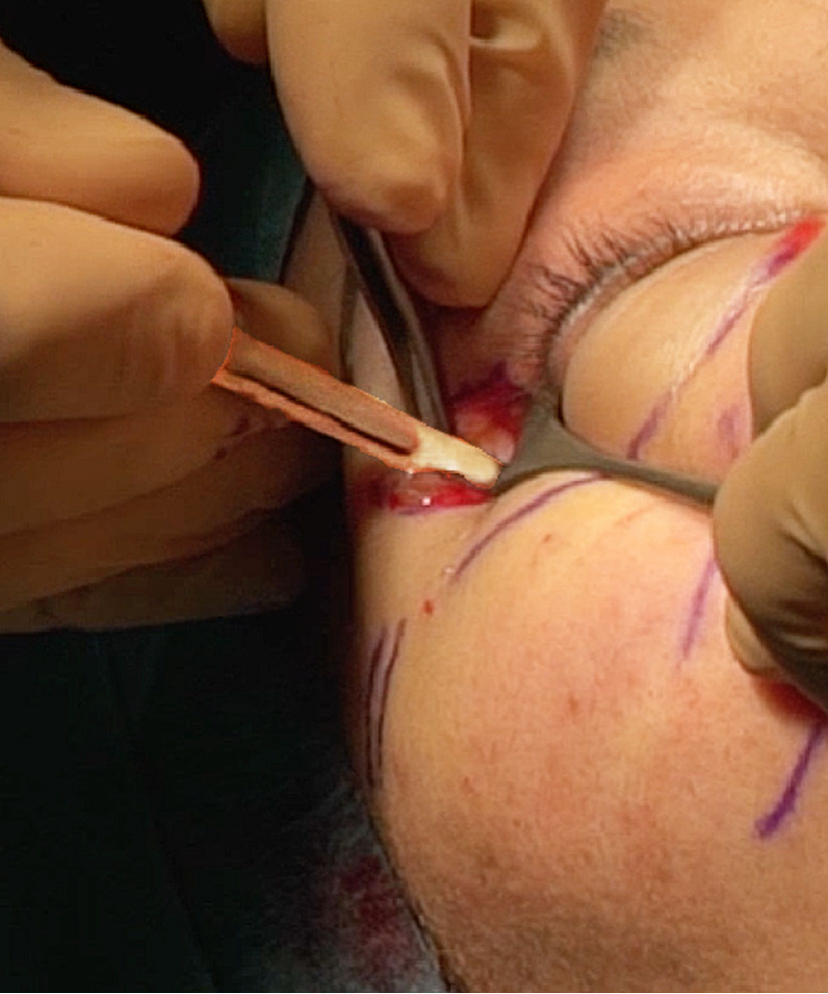
Frequently, subperiosteal dissection begins through an upper-eyelid opening with a scalpel 15 blade to avoid a dissector sliding

Upper-eyelid surgery is frequently also undertaken and, if so, the subperiosteal dissection begins through the lateral part of the upper-eyelid incision. If no upper-eyelid surgery is performed, a specific 2-cm-long upper-eyelid incision above the lateral canthus is required to be able to perform the dissection of the lateral and inferior orbital rim, which is mandatory for the canthopexy (Fig. 4). This scar fades rapidly. The subperiosteal dissection through the upper-eyelid incision starts with a 15 scalpel blade to incise the periosteum. Dissection is continued with the use of a 10-mm Obwegeser elevator in the malar area.

### Subcutaneous Lower Eyelid Dissection

The subciliary eyelid incision extends from the lacrymal point to 4 mm outside the lateral canthus.

The height of the subcutaneous dissection is the height of skin to be resected in each case, as previously explained:

1. 10–25 mm, if no previous surgery.
2. 5–15 mm, if previous lower eyelid surgery with no eyelid retraction.
3. 0 and 10 mm in case of lower eyelid retraction due to previous surgery.

### Lower Eyelid, Subperiosteal Dissection

**Fig. 5:**
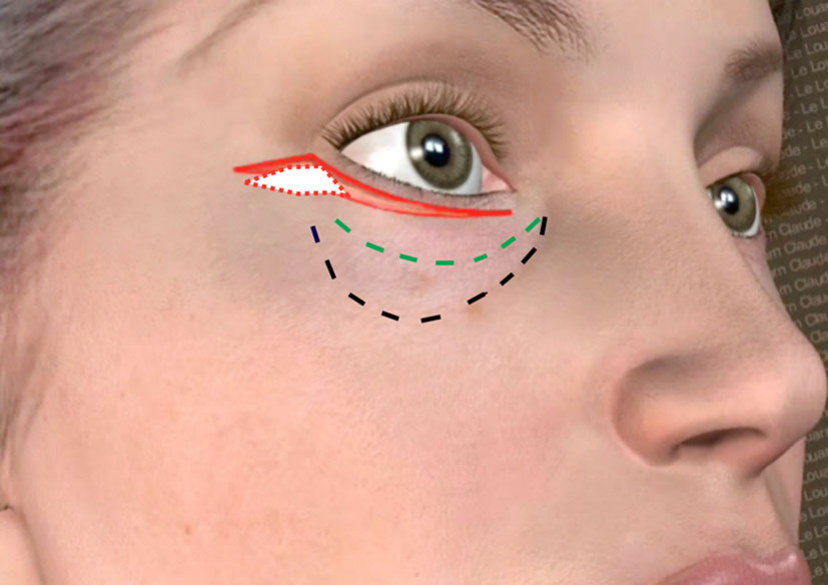
The 2-cm muscle opening at the lateral canthus allows the subperiosteal dissection, thanks to palpebral tissue laxity. The subcutaneous dissection is half the distance to the lid-cheek junction

The subperiosteal dissection begins with a 2-cm incision through the orbicularis muscle (Fig. 5) at the lateral canthus, following the lateral extremity of the subciliary incision. To avoid the risk of secondary eyelid malposition, muscle incision has to be 15-mm infero-lateral to the lateral canthus and only 5 mm horizontally medial to the canthus. Through this 2-cm opening, the lateral and inferior orbital rims are freed subperiosteally.

**Fig. 6:**
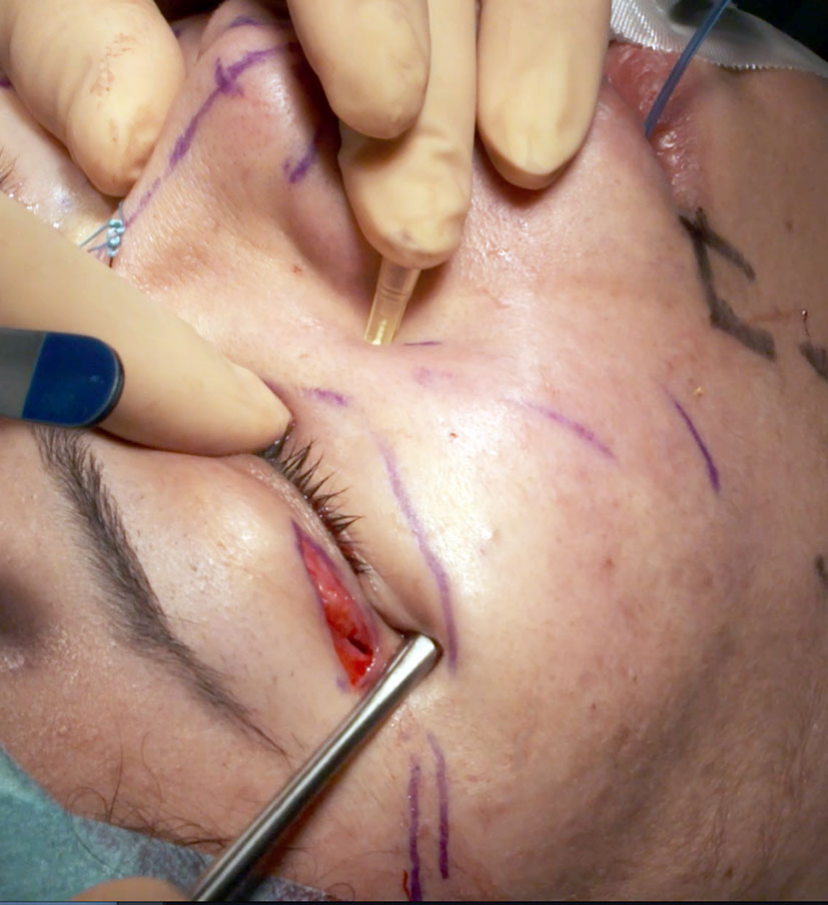
If localization of the infra-orbital foramen with a 23- or 28-G needle is easy, subperiosteal dissection can be performed through the lateral palpebral opening. Otherwise a buccal opening is necessary

Then, the dissection is continued through the anterior malar area. At this stage, the infra-orbital nerve must be taken into account, because the muscular incision is purely lateral and does not provide visibility of the paramedial malar area and the infra-orbital nerve. To prevent damage to the infra-orbital nerve there are two options:

1. Perform the dissection, under direct vision, through an upper buccal sulcus incision,
2. Use a thin 23-G or 28-G needle (too thin to really damage the nerve) passed through the skin into the infra-orbital foramen to locate the origin of the nerve (Fig. 6). Nevertheless, to minimize the risk of transitory nerve damage with a 23-G needle, a mandrel with a diameter of 0.5 mm can be used, just after the skin puncture with the needle, to locate the foramen. The dissection is then performed around this marked area. When the location of the infra-orbital foramen is difficult to determine precisely, it is mandatory to return to the first option and to identify it through the upper buccal opening.

Subsequently, the dissection is continued medially and ends at the surface of the nasal bones area above the infra-orbital nerve. Disinsertion of the medial part of the arcus marginalis must be limited to reduce the risk of damaging the lacrymal sac. In between the arcus marginalis and the infra-orbital nerve, the palpebral part of the orbicularis oculi, the tear trough ligament and the orbital part of the orbicularis oculi are released. An intra-orbital dissection of 1 cm is necessary all along the lateral and inferior orbital rim in order to drill 6 holes for fixation.

A curved 10-mm Obwegeser dissector needs to stay positioned lateral and below the infra-orbital nerve, toward the pyriform aperture, in contact with the bone to protect the inferior branches of the infra-orbital nerve.

Laterally, the extent of the dissection releases the body of the zygoma but does not need to reach the arch of the zygoma. Inferiorly, to detach the mid-face flap completely and obtain an effective upward vertical movement of the tissues, a release of periosteal insertions at the inferior malar border is performed with a 20-mm elevator. The dissector is used gently and is passed vertically downward into the cheek (the opposite of a Gillies lift) elevating malar tissues, to give better feeling of the periosteal resistance. This dissection is performed gently to avoid bleeding: this is the only area where bleeding may be a problem, at the junction between the non-vascularized subperiosteal area and the vascularized cheek [15].

### 3 Pairs of Drill Holes Per Side

The holes are 1.5 mm diameter, and for each pair, there is 3 mm between the two sets of holes. They are numbered from 1 to 6 from medial to lateral, in a clockwise direction.

**Fig. 7:**
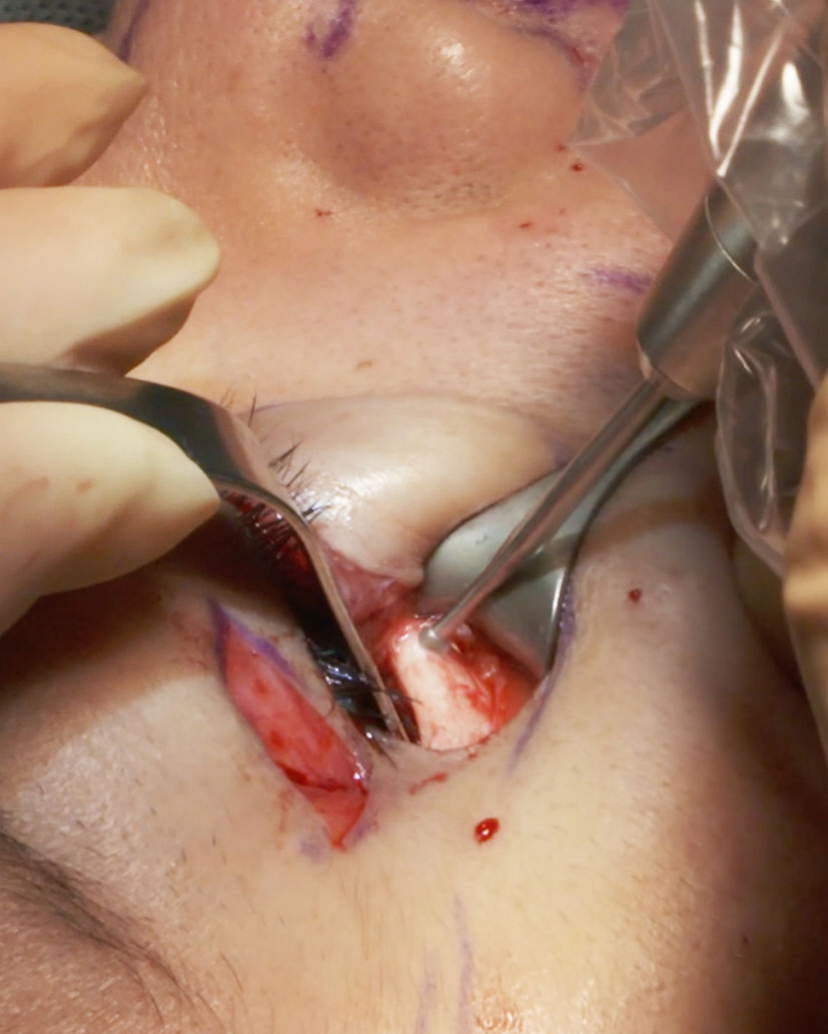
Hole n°1 is drilled the more medially possible, on the inferior orbital rim, near the mid-pupilla line

**Fig. 8:**
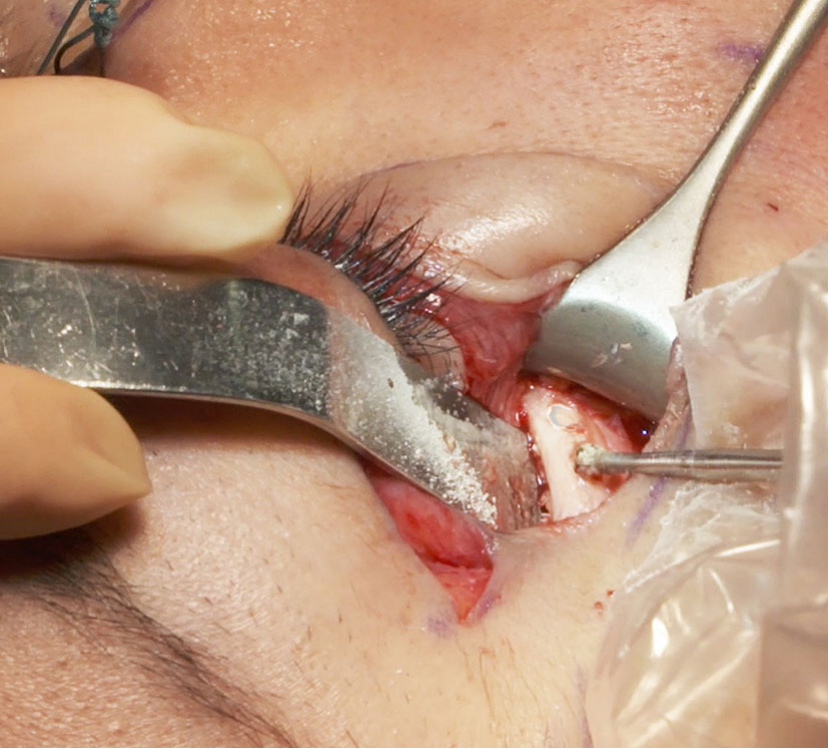
Like the other drill holes 3 and 5, drill hole n°2 is three mm apart from the previous one

**Fig. 9:**
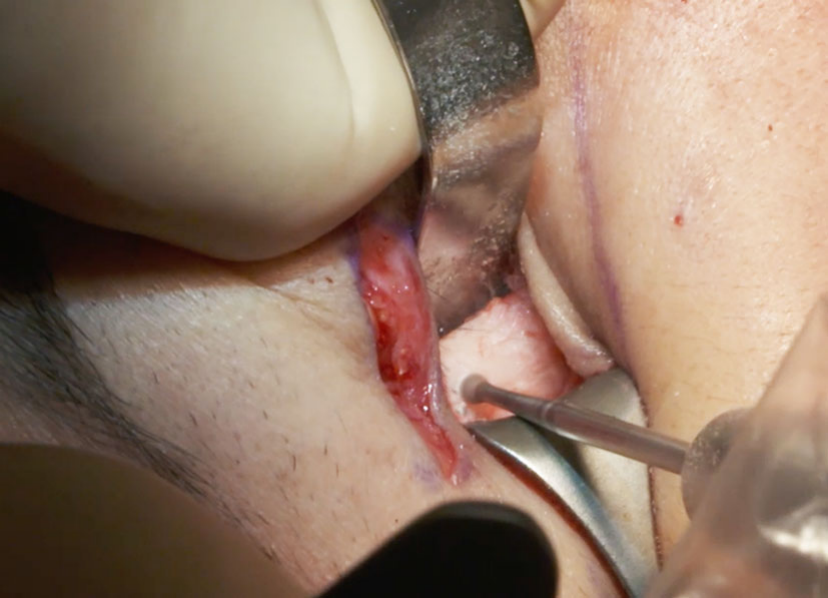
Hole n° 4 is located just below the lateral canthus

**Fig. 10:**
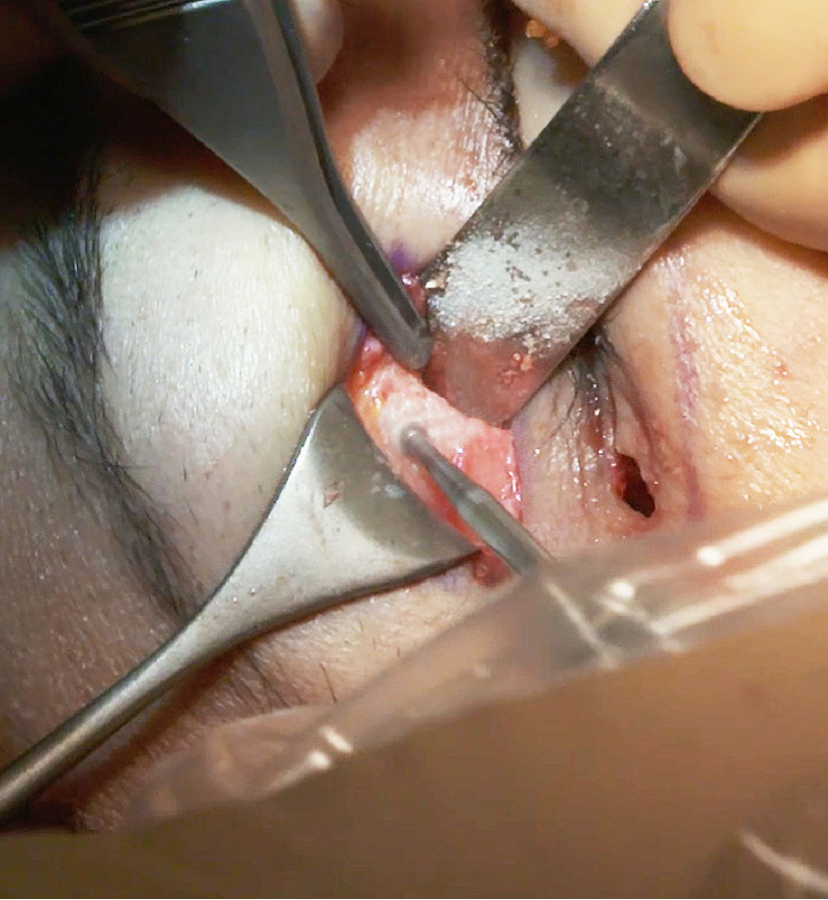
Hole n°6 is directed backward inside the orbit. As it will be the only point of traction of the tarsus extremity, the position backward inside the orbit maintains the tarsus on the globe

The first pair of holes is drilled as medially as possible on the inferior orbital rim in the mid-pupilla line, depending on the skin and muscle elasticity in the area of the incision (Figs. 7, 8), thanks to the complete subperiosteal release of the lateral and inferior orbital rim. The second pair is drilled at the lateral orbital rim (Fig. 9), below the lateral canthus, and the last pair is at least 1 cm above the lateral canthus to perform a symmetrical canthopexy. The upper one is directed backward inside the orbit (Fig. 10), to avoid creating a gap between the tensed tarsus and the globe. Thus, the tarsus will follow the shape of the globe.

### In Case of Prominent Postseptal Fat Pads, the Sliding Fat Pad Technique is Used

The technique was first described by Loeb and updated by de la Plaza [18], using the transconjunctival approach. If lower lid fat pads are associated with a tear trough, the inner and central fat pads are used, as fat flaps, to fill the underlying depression, after releasing the tear trough ligament.

Incision of the conjunctiva and the capsulopalpebral fascia is performed about 2 mm inferior to the lower edge of the tarsus.

Dissection [19] is performed following the preseptal–suborbicularis dissection plane (not opening the septum), freeing subperiosteally the arcus marginalis to see the inferior orbital rim. Dissection stops subperiosteally and develops more anteriorly and superficially with the visualization of fibers of the orbicularis oculi and of the levator labii superioris, which herald the entrance into the premaxillary space and accordingly the complete release of the tear trough ligament.

The orbital septum is opened, and blunt dissection of the inner and central fat pads is performed. The fat flap is secured with a transcutaneous stitch tied over a small piece of gauze. The correct positioning of the fat flap below the tear trough, into the premaxillary space, is checked. Two transconjunctival Vicryl 6-0 rapid sutures can close the mucosal opening.

### Spacer Graft

If a deficit of the posterior lamella has been detected preoperatively, the insertion of a spacer graft is mandatory. The author prefers using hard palate/mucosal grafts instead of biological implants like Permacol (Covidien, Dublin, Ireland).

The advantage of the hard palate/mucosal grafts is that it can be used even in case of patients with risk factors for contracture, poor vascularization and inflammation, conditions which are frequent in the surgical treatment of secondary eyelid malposition [20].

The mucosa is harvested paramedially, usually 5 mm high and 25 mm in length for one lower eyelid. The knife does not go deeply until the bone but stays just below the mucosal level to avoid any postoperative discomfort.

**Fig. 11:**
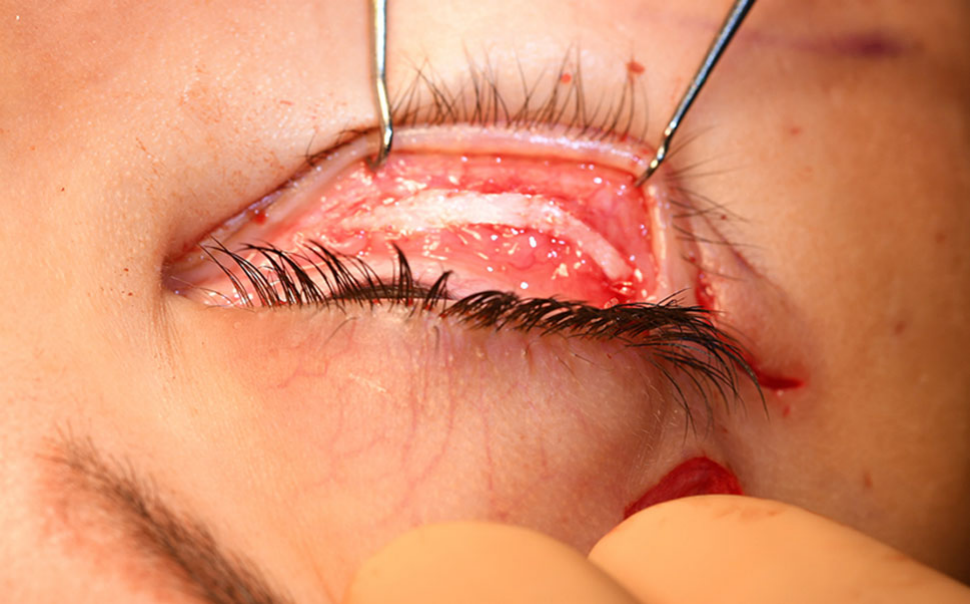
This spacer of 3 mm height will participate with a concentric malar lift to elevate 2 mm of the lower lid margin in an aesthetical indication

It is positioned through a horizontal transconjunctival incision in the precise location of maximum retraction: the conjunctiva and lower lid retractors (capsulopalpebral fascia) are incised, and the spacer is placed into the opening to lengthen vertically the posterior and middle lamella (Fig. 11). A Vicryl 6-0 rapid suture is used to attach the graft to the recipient conjunctiva and capsulopalpebral fascia [8].

### Malar Elevation and Concentric Fixation

**Fig. 12:**
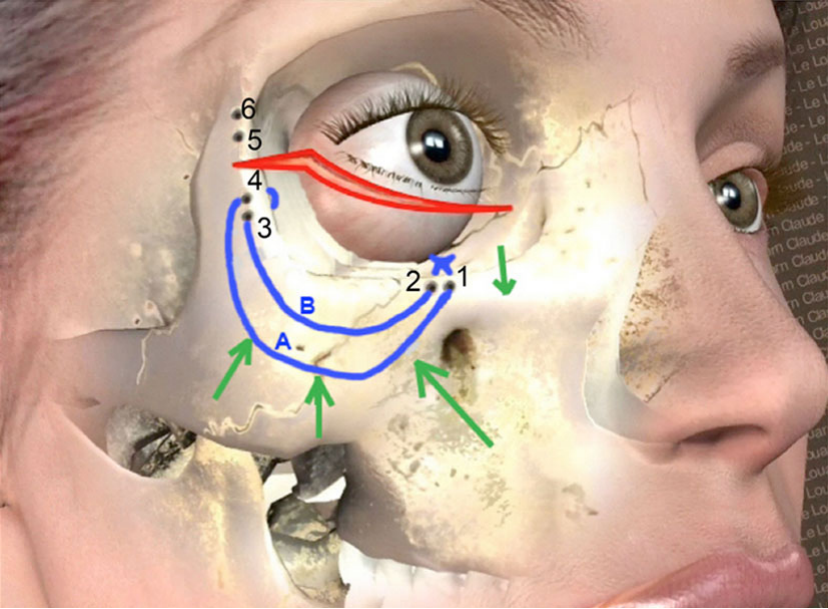
Passing the thread A through holes n°4 and 1 elevates the lower malar area. Passing the thread B through holes n°3 and 2 elevates and flattens the malar mound. On this drawing, the thread is passed through holes n°3 and 4 from inside to outside

Through hole n°4, below the lateral canthus (Fig. 12), one of the two needles of a polypropylene Quill suture 2/0, 7 × 7 cm configuration (Angiotech Pharmaceuticals, Inc.) is passed from intra-orbital to extra-orbital and bites taken through the periosteum, the lower lateral malar area, which is simultaneously elevated with a Gillies hook [15]. The choice of this barbed suture is just a personal preference. The tip of the needle is directed toward the malar bone, and a forceps can easily catch it for a second bite. This second bite continues the horizontal elevation of the lower malar area through the fat of the upper part of the nasolabial fold, which is elevated with a Gillies hook. After this bite, the needle enters inside the orbit through hole n°1 and this first pass is tensed, producing an efficient ‘en bloc’ malar elevation.

Of course, care must be taken not to create a major skin depression during this concentric elevation of the lower part of the malar area. A small depression is normal and will fade in 15 days. To minimize the risk of skin depression and the risk of muscle (orbicularis oculi, zygomaticus, levator labii, etc.), or motor nerve damage, the needle needs to bite the periosteum, the suborbicularis oculi fat but must not go more superficially to avoid any functional damage.

Through hole n°3, which is located 3 mm below hole n°4 on the lateral orbital rim, the second needle of the Quill suture is passed from intra-orbital to extra-orbital and bites horizontally the lower and deep part of the malar mound. After this bite, the needle enters inside the orbit through hole n°2. The thread is tensed, inducing an efficient malar mound elevation and flattening. The horizontal malar depression created by this pass will fade thanks to the following orbicularis muscle elevation, produced by the canthopexy. The ends of the two threads are knotted inside the orbit.

### The Lateral Canthopexy

Because the lateral canthus insertion on the rim has already been released subperiosteally, a canthopexy is necessary in all cases to stabilize the canthal position. When a change of eye shape is planned preoperatively, the canthus can be positioned more laterally and/or higher.

A lateral transosseous and transcartilaginous canthopexy is performed between the lateral extremity of the lower tarsal plate and the lateral orbital rim (drilled holes n°5 and 6). It should be noted that a transperiosteal canthopexy is never used by the author because it cannot compete against the strong orbicularis muscle contraction during the blinking reflex in the lateral canthus which results in it being transposed 4 mm medially.

A Vicryl 5-0 is passed through hole n°6 from outside, and the needle bites the lateral edge of the tarsus from outside and then bites again from inside to produce a solid ‘U’ bite of the tarsus. The thread is again passed through hole n°6 but from inside and goes through hole n°5 and is then tied. Note that the suture goes and returns through the same hole (n°6) to give a precise and posterior point of traction at the extremity of the tarsus, following the curve of the globe.

**Fig. 13:**
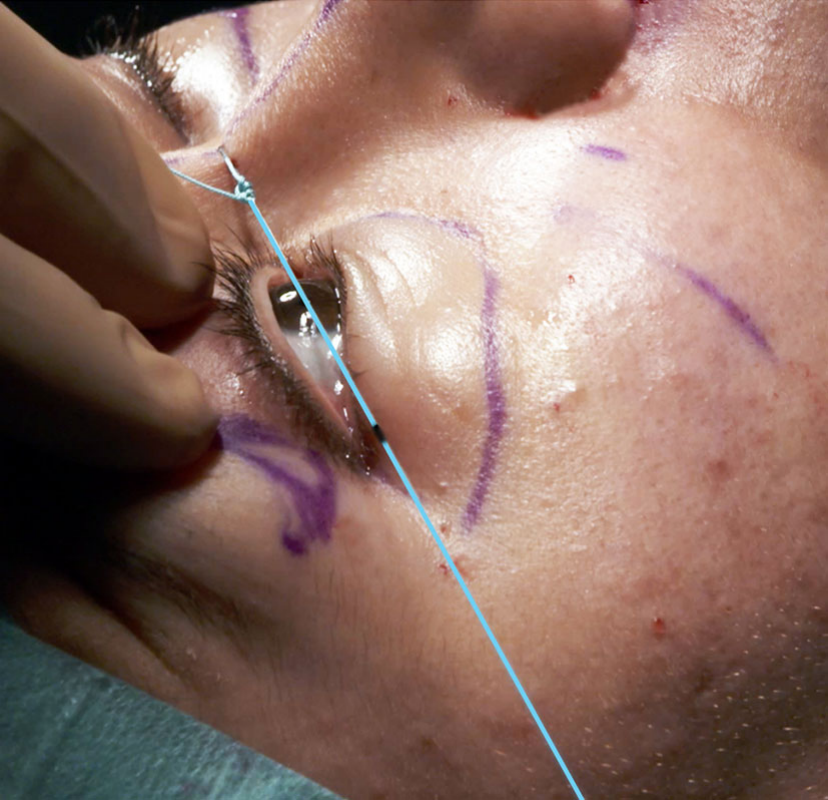
This thread is tensed from the root of the nose, at the medial canthus height, toward the lateral canthus. A mark is drawn on the projection of this thread in the temporal area. A dark mark locates the position of the lateral canthus on the thread to determine the importance of its lateral transposition, if necessary

To determine the precise location of the canthus, a thread is fixed at the root of the nose, at the height of the medial canthus (Fig. 13). This thread is tensed through the lateral canthus toward the posterior temporal area. The place of the thread in the posterior temporal area is marked. This marking is always made in the posterior temporal area to prevent any error if an anterior temporal lift is performed as well. When a lateral transposition of the canthus is planned, a second mark is placed on the braided thread with a marker to determine the precise position of the canthus on the thread before surgery.

During the surgery, the canthus is usually replaced on the same line and location as agreed with the patient, even though with age the canthus has drifted slightly medially.

Even if the canthopexy is planned to change the canthal position, it is still a Vicryl 5-0 which is used to anchor the canthus through the bone in its new position. If a permanent thread was used, 1 year later, it would appear subcutaneously because the tarsus fixation would have worked loose. To avoid this medium-term problem and achieve a stable new canthal position, botulinum injections are used to stop any orbicularis muscle movement for 3 months. This immobility permits healing to occur without any muscular deforming force and will produce a stable result.

In cases of cantonal elevation, to ensure symmetry, measurements of the vertical distance between the new position of the canthus and the thread joining the nose to the temporal mark are performed.

In case of lateral transposition, to ensure symmetry, measurements between the mark on the thread and the new canthal position are performed. In the author’s experience, these are the simplest and fastest ways to determine symmetry in height and width of the canthi.

### The Orbicularis Oculi Muscle Suspension

A last suspension is performed between hole n°6 (Fig. 9) and the orbicularis oculi muscle to avoid any secondary muscle retraction. The Vicryl 5-0 needle goes through hole n°6, descends submuscularly and reaches the opening in the orbicularis oculi. A solid bite in the orbicularis oculi muscle is taken at the inferior border of the subcutaneous dissection.

The thread returns submuscularly to hole n°6 and is tied.

This vertical suspension must not induce any elevation of the canthal position as previously determined. Overcorrection has to be avoided.

Performing the lateral canthopexy in this way will eliminate the risk of secondary descent of the posterior lamella, and performing the orbicularis oculi muscle suspension will eliminate the risk of secondary descent of the anterior lamella.

### Skin Removal

This en bloc and ‘hammock’ double concentric elevation of the malar area produces major lower eyelid skin excess, especially in patients who have had no previous surgery. Frequently, half of the distance between the eyelashes and lid-cheek junction is removed. Ten to 25 mm of ‘tensed’ skin height is frequently removed, but, of course, this does not represent 2 cm of non-tensed lower eyelid skin and the thick skin of the lid-cheek junction is never attached to the eyelash margin because a large part of the eyelid skin is kept.

As previously explained, the main skin excess treated with this technique is in the mid-pupilla line, which corresponds to the exact location of the major skin excess in the underlying malar area. At the extremities, on the medial and lateral canthus, the skin excess is smaller. At the medial canthus, the complete skin excess is removed without risk. At the lateral canthus, the complete skin excess must be preserved to avoid ectropion. Postoperatively, this lateral skin excess is always resorbed naturally in 24 h (which explains why its removal would lead to eyelid malposition).

Thanks to the new definitive adhesion between the elevated periosteum and the malar bone and thanks to the strong stability of this double, concentric thread elevation, the skin excision is particularly safe.

**Fig. 14:**
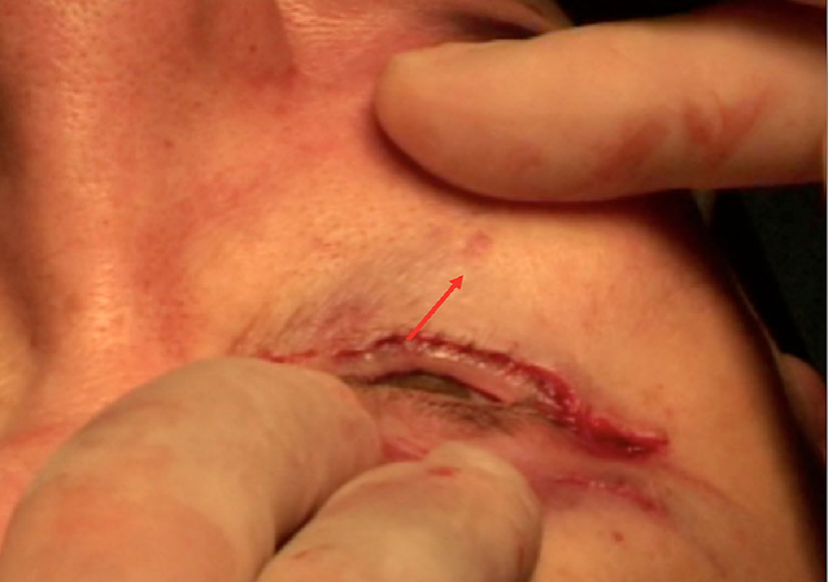
The lower eyelid skin excision must be controlled to avoid any lower eyelid margin descent during downward traction of the malar area

To remove the skin excess, two vertical incisions like visible in (Fig. 3) are distributed through the skin to be removed and two Vicryl 6/0 rapid fix the lower extremity of each vertical incision to the horizontal lower eyelid incision. To check the height of resection, downward traction is applied on the malar skin with a finger and the lower eyelid margin must not descend (Fig. 14).

If this downward traction induces lower eyelid descent, less skin is removed in these three vertical lines. With experience, if this traction is far from mobilizing the lower eyelid, more skin can be removed. Finally, the established skin excess is excised and some more Vicryl 6/0 rapid sutures are inserted to close the incision. Frequently, more skin than planned can be removed near the medial canthus and, as stated, no skin has to be removed at the lateral canthus.

Of course, at the beginning of the learning curve, less skin than that which was recruited is excised. Keeping a safety margin of 3 mm of the 20 mm to excise is recommended at this stage. If necessary, a strip of skin excess can be removed under local anesthetic once healed.

This technique has the advantage of preserving the orbicularis oculi muscle, which never needs to be excised. Muscle function is completely preserved, and only the skin dissected off the orbicularis oculi muscle is removed.

### Postoperative Period

Because the only dissection plane is subperiosteal and the tissues are strongly attached to the bone with the barbed sutures, swelling remains acceptable for a mid-face lift. The subcutaneous dissection plane corresponds to the skin excision, and so no longer exists at the end of the procedure which limits most of the risks of subcutaneous dissection (necrosis, irregularities). However, the use of a lateral canthopexy can produce temporary chemosis. Chemosis developed in 31 patients (11%). This settled in all cases within a month through the application of a steroid eye drop four times a day for 2 weeks, cold compresses and specialist lymphatic drainage. A layer of thick cream was also applied to protect the eye during the night. Refractory chemosis can be treated with a cortisone injection (acetonide of triamcinolone, 1/10 of a ml diluted 2 times), in the preperiosteal zone, at the inferior extremity of the mid-cheek furrow, where deep and superficial lymphatics of the lower eyelid converge [21].

Avoiding working on a computer screen is mandatory for 2 weeks following surgery. Pain is rare and limited. In many cases, patients have difficulties in dealing with their postoperative surgical appearance and require more postoperative consultations than in most other procedures. In most cases, this lasts for 2 months and in some cases it can take up to 4 months.

## Results

This study has been carried out on 342 patients (684 eyelids) who underwent concentric malar lift with barbed sutures from 2010 to 2016. A total of 256 cases (75%) were aesthetic mid-face lifts, whereas 86 cases (25%) were for lower eyelid retraction. A spacer graft was used in 30 of these 86 cases (35%).

Two hundred thirty patients in the study had upper-eyelid surgery performed in addition to their concentric malar lift, and 132 had a facelift associated.

Only 10 of the patients underwent canthopexy with lateral bone removal to achieve a more lateral position of their canthus for cosmetics.

A concentric malar lift was recommended to 9 patients because of dissatisfaction with their previous standard lower eyelid surgery.

For aesthetic patients, improvement was measured and analyzed for the study on the pre-op and postoperative pictures and was directly related to the height of skin removed as measured and photographed during surgery. The amount of skin available for removal is the direct consequence of efficient malar elevation.

Patients with preoperative lower eyelid descent frequently have associated functional problems, and evaluation was carried out through analysis of both the questionnaires and pictures. The concentric malar lift outcomes were considered satisfactory by nearly all these patients. The patients highly valued their aesthetic improvement combined with the marked improvement of their ocular symptoms (Table 1).

Symptoms, such as excessive tearing and blurred vision, needed nevertheless 1 year to settle completely, whereas others, such as ocular discomfort, while less measurable and more variable, were reduced considerably (Table 1). Moreover, the associated mid-face lift also results in cosmetic improvement of the entire region of the eyelid and mid-face. Two patients who underwent concentric mid-face lifts, despite psychological problems, because they had major eyelid descent and associated ocular problems, did complain about the absence of any improvement aesthetically and physiologically—regardless of an objective improvement in lower eyelid position as shown in their pre- and postoperative pictures!

**Fig. 15:**
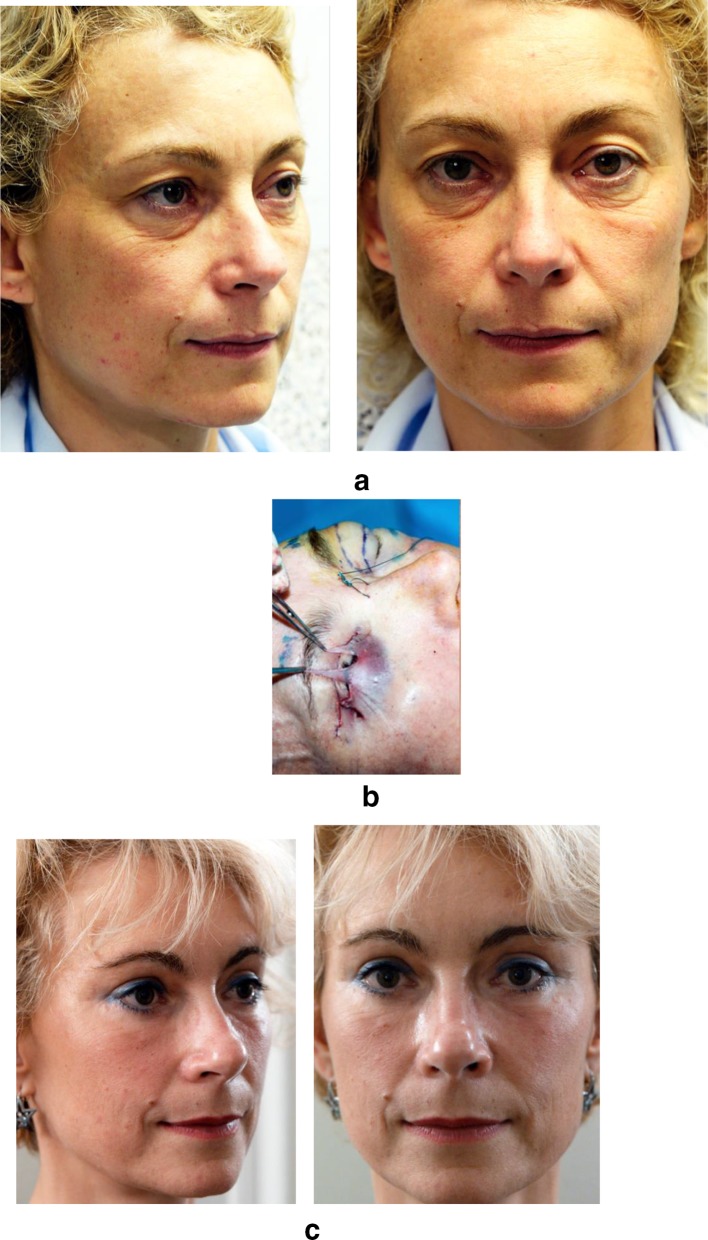
**a** A 45-year-old patient with upper and lower lid skin excess and presenting a corrugator with elevated resting tone. **b** Vertical skin excess (2 cm) was removed at the mid-pupilla line with no lateral skin excision. **c** Six months after upper blepharoplasty with corrugator muscle weakening and concentric malar lift. The eye shape looks natural
- Case 2 (Fig. 16) is a full face rejuvenation, associating a concentric malar lift (2 cm of skin removed) with upper blepharoplasty, corrugator muscle weakening, neck lift with platysma fixation to the deep cervical fascia (Hyo-neck lift evolution), DAO weakening, lip lift with VY flaps and rhinoplasty, in the same session.

Five cases are illustrated:

- Case 1 (Fig. 15) is a classic, cosmetic concentric malar lift for facial rejuvenation.

**Fig. 16:**
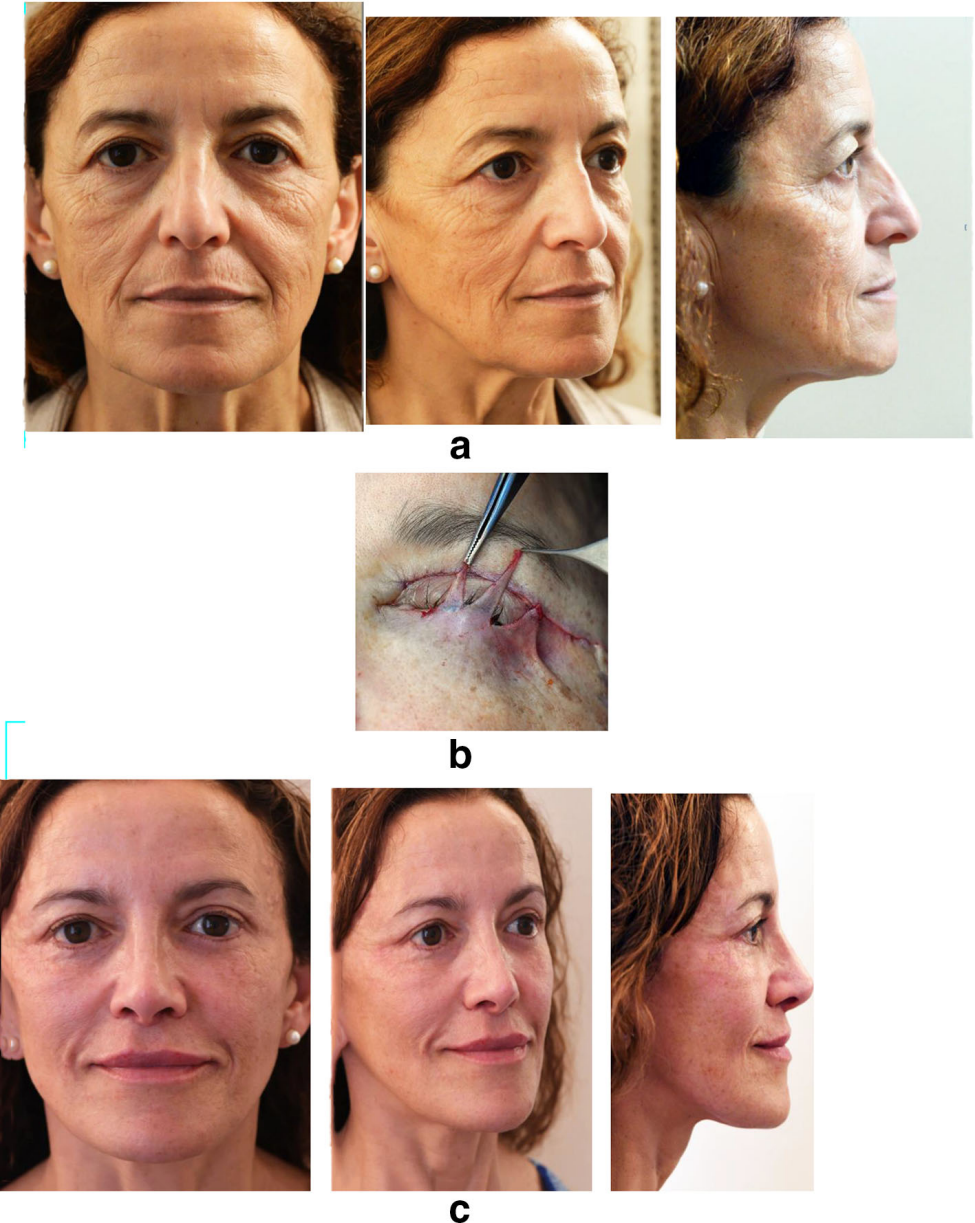
**a** A 58-year-old patient with upper-eyelid and mid-face skin excess. **b** An upper blepharoplasty was performed with a corrugator muscle weakening, associated with a concentric malar lift (2 cm of vertical skin removed), a neck lift (platysma to deep cervical fascia fixation) with DAO weakening, a lip lift with VY flaps and a rhinoplasty, in the same session. **c** The postoperative eye shape is identical to the preoperative appearance
- Case 3 (Fig. 17) illustrates the treatment of a congenital lower eyelid malposition with an important negative vector. The concentric malar lift was performed with a spacer.

**Fig. 17:**
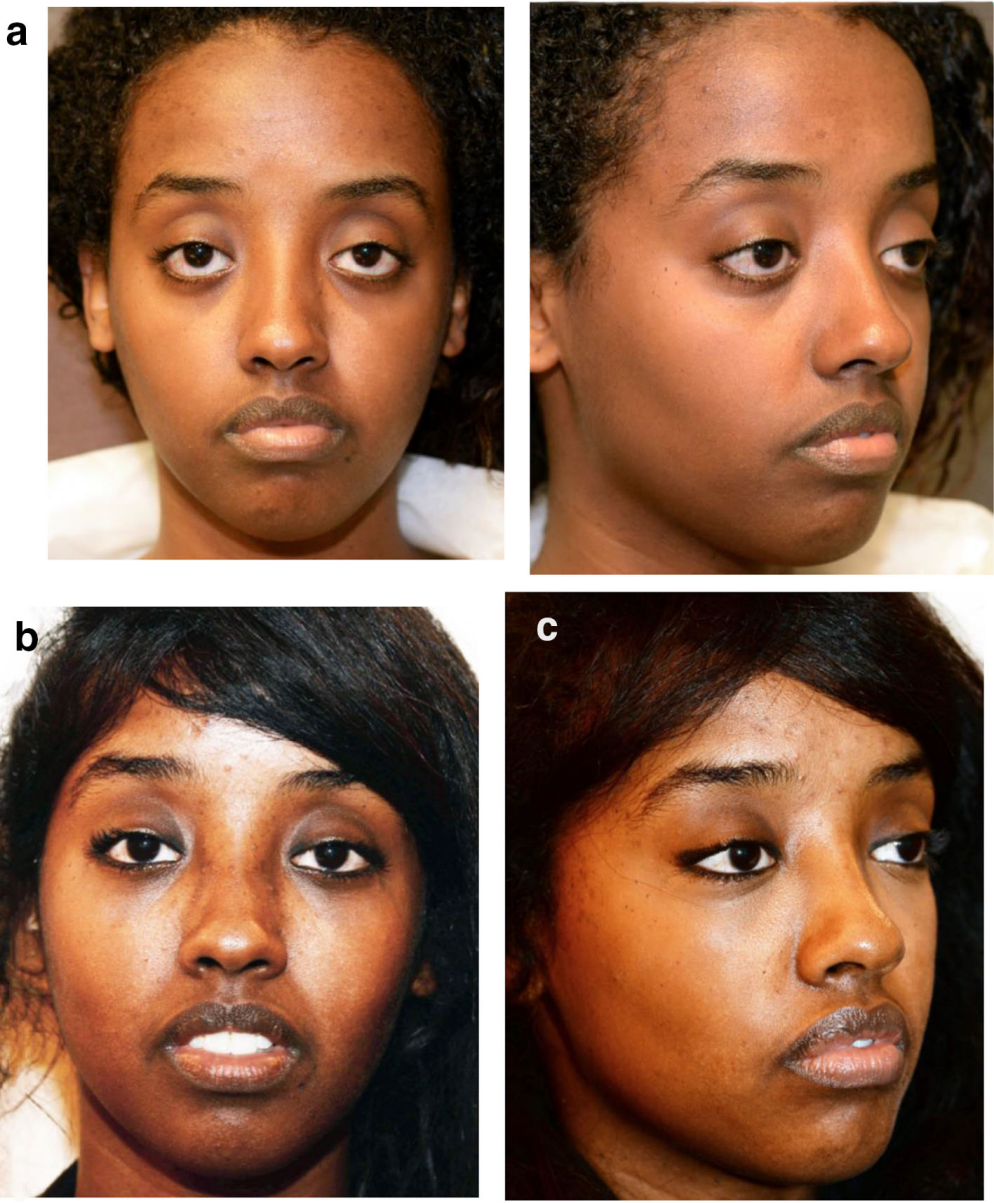
**a** Without malar support, 20-year-old patient presenting with congenital descent of the lower eyelid, of the lateral canthus. **b** A concentric malar lift and a hard palate/ mucosal graft of 4 mm each side were performed without any fat injection: the elevation of the malar fat pad on the malar bone induced sufficient volume. **c** Aspect at 1 year post-op with a correct and stable lower eyelid elevation
- Case 4 (Fig. 18) had an ectropion following aesthetic blepharoplasty treated with a concentric malar lift and a spacer.

**Fig. 18:**
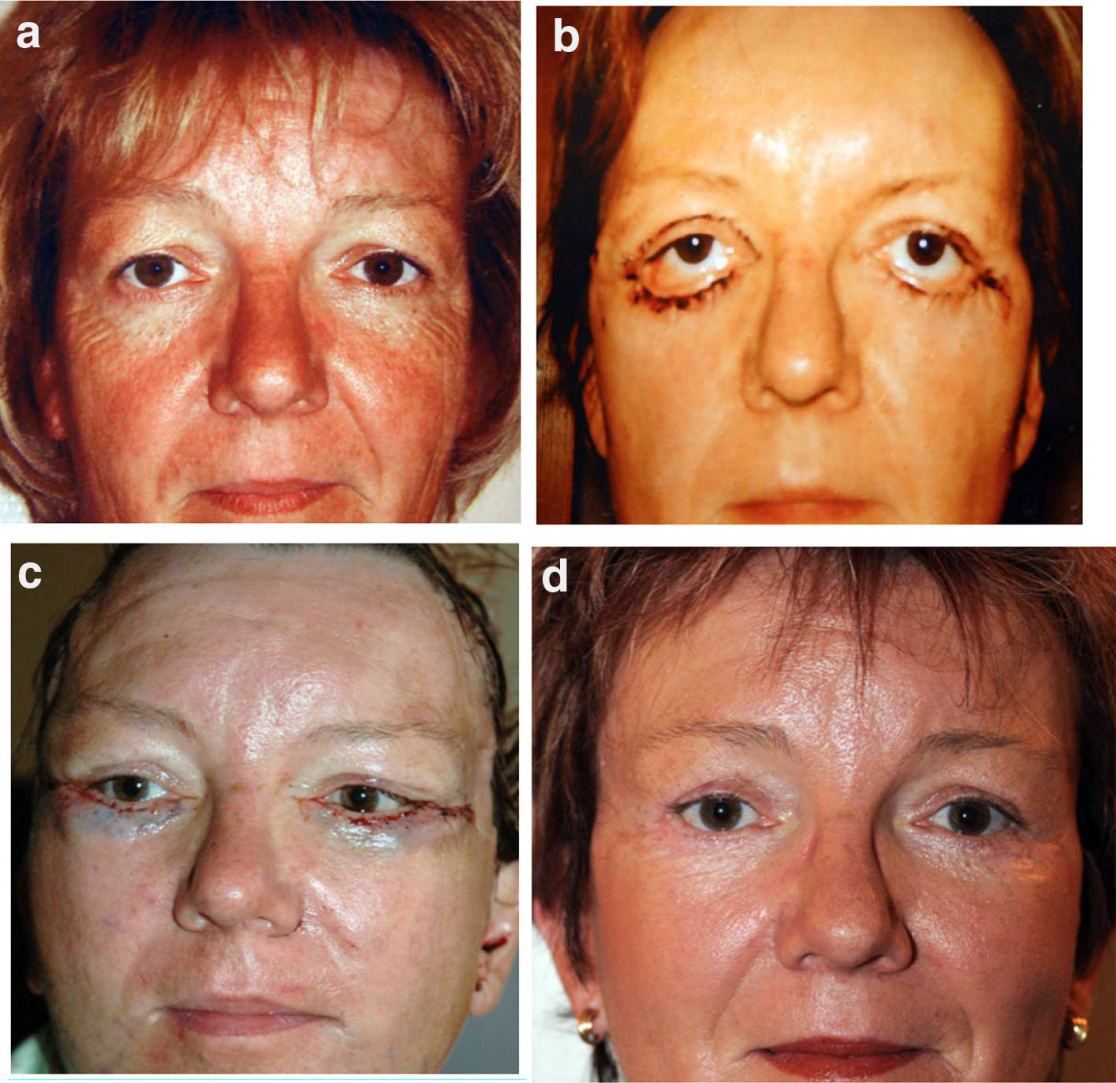
**a** Preoperative view before a standard blepharoplasty. **b** Postoperative after a standard blepharoplasty with bilateral ectropion. **c** One day post-op after a concentric malar lift and a spacer: correction of the ectropion is achieved. **d** The result at one year post-op demonstrates the good stabilization of the lower lid elevation
- Case 5 (Fig. 19) was a severe case of lack of skin at the lateral canthus level due to 2 previous operations and complicated by a malar area inflated with Bio-Alcamid which had become infected.

**Fig. 19:**
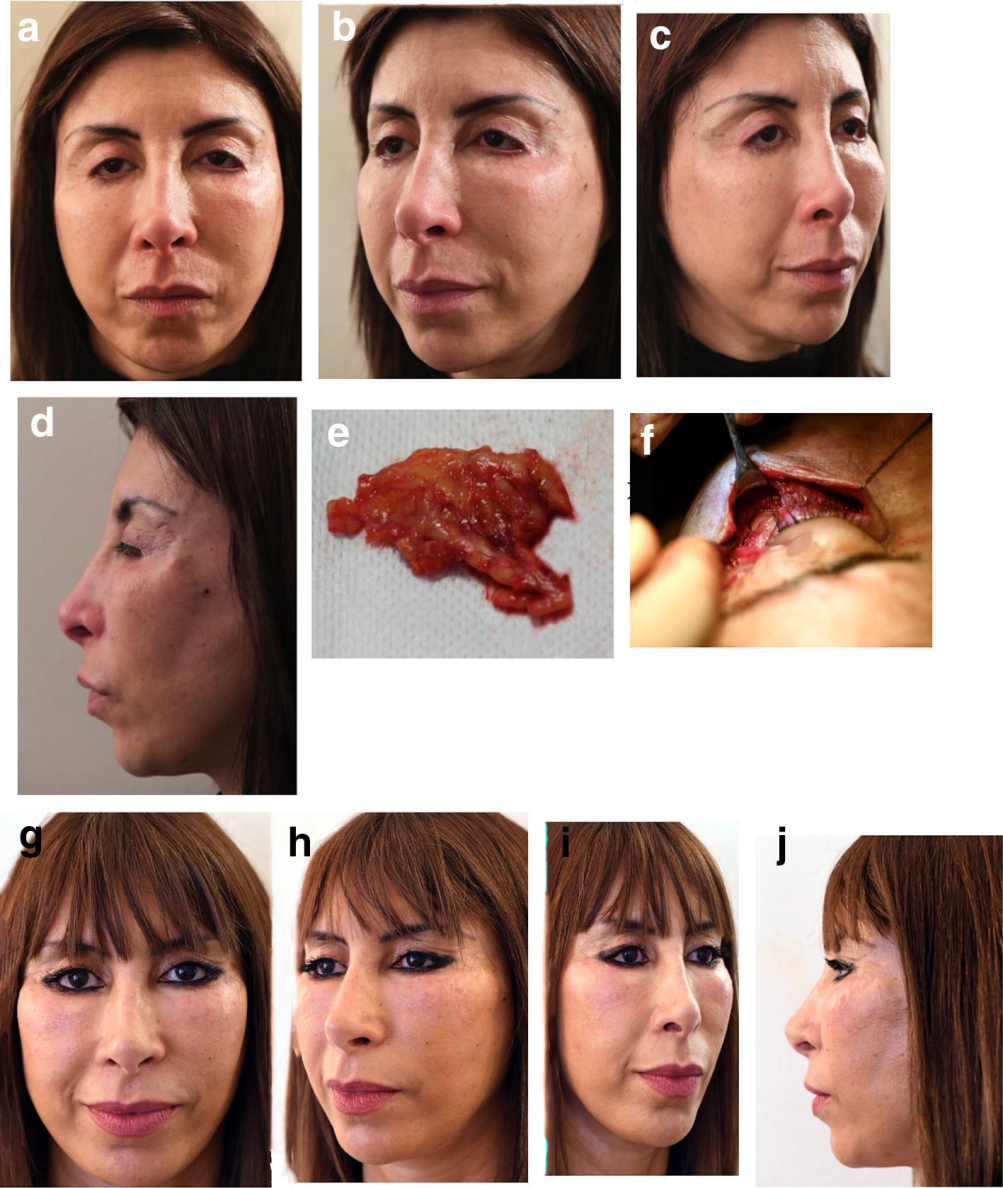
**a**, **b**, **c**, **d** A 53-year-old patient, operated on 2 times for blepharoplasty, with a dramatic descent of both canthi and of the lateral part of the lower eyelids, due to a major loss of tissues. At the end of his procedure, the previous surgeon tried to elevate the lower eyelid with the injection of a large quantity of Bio-Alcamid in both malar areas. This unfortunately induced inflammatory reactions during the last 2 years. Decision was to remove the maximum of Bio-Alcamid, nevertheless trying to preserve the innervation and mobility of the lower eyelid (**e**). After removal of blocks of Bio-Alcamid, only partial flaps of muscle were still there. **e** A block of Bio-Alcamid removed from the malar area. **f** After removal of Bio-Alcamid, only partial flaps of muscle were still there. **g**, **h**, **i**, **j** A concentric malar lift was used to stabilize the mid-face ascension with a spacer placed on the whole lower eyelid length, from 4 mm height medially to 15 mm height at the lateral canthus level. The patient asked for not using any skin graft but agreed for a classical lateral skin/muscle upper-eyelid flap. The quality and the function of the result were surprising after such a preoperative complicated case and such a major surgical traumatism

These cases illustrate the adaptability of the concentric malar lift technique for all mid-face indications, from standard lower eyelid rejuvenation to the worst case of reconstruction.

### Complications

On the 342 cases studied, the following complications and sequelae were encountered.

Two patients (0.06 percent) reported decreased sensation in the distribution of the infra-orbital nerve on one side. These settled spontaneously in 4 months.

Six patients (1.8%) developed detachment of the lateral canthus at 4 weeks post-op, due to excessive scarring. All of them had undergone a previous unsuccessful surgery of the area (mid-face lift, canthopexy, spacer, etc.). All had been counseled about the risk of complications. No further operation was required, and each was treated successfully with the deep injection of 0.05 ml acetonide of triamcinolone diluted 1 time in the area of maximum retraction and also with the use of upward massage of the eyelid margin, 4 times a day for 2 months, under the direction of a physiotherapist. The massage progressively elevates and disinserts the eyelid margin at the lateral canthus. Good support from both the surgical team and the physiotherapist is mandatory in the healing period.

## Discussion

The first part of the discussion covers the indications for the concentric malar lift in cases of lower eyelid and mid-face aging, compared to other surgical rejuvenation techniques (e.g., standard lower eyelid surgery with fat grafting, other types of mid-face lift).

The second part discusses the role of concentric malar lift in reconstructive cases (congenital lower eyelid or post-traumatic descent), compared to other surgical reconstruction options (skin graft, local flap or other mid-face lift techniques).

### I/Aesthetic Discussion

#### Comparison with Other Surgical Rejuvenation Techniques

- Fat grafting versus concentric malar lift

In aesthetic rejuvenation, it may be more attractive for the surgeon and the patient to choose fat grafting of the nasojugal furrow, malar area, mid-cheek furrow instead of performing a mid-face lift as this solution appears much easier and less risky to the patient and the surgeon. Tonnard [5], who is well known for his experience in the technique, wrote he prefers micro-fat grafting to mid-face rejuvenation techniques. In the same way, Rohrich [22] explained that for mid-face rejuvenation, he performed ‘selective fat compartment filling of deep malar and high malar locations and nasolabial fold fat grafting to precisely control facial contouring.’

If the facial improvements induced by fat grafting are unquestionable, the question is to what extent this improvement constitutes a rejuvenation or a change of the face.

Is this change of the face desired or acceptable for the patient? The author has not found any publications reporting scanner or MRI studies proving the existence of mid-face fat depletion with aging even in publications supporting fat injections as the solution for mid-face rejuvenation.

To the contrary, Jang [23] concluded in his work referenced below ‘This study suggests that the mid-facial fat pad is thickened in the elderly.’ And he is not the only one, and many other 3D studies of the mid-face [24–26] conclude that there was no mid-face fat deflation with time.

Of course, fat injection and fillers can successfully hide a depression due to a fat transfer, but because there is no mid-face deflation with time, volume augmentation is only a compromise and a puffy look must be avoided at all cost. In the mid-face, as well as in the rest of the face, when skin excess occurs, only skin removal and fat relocation can rejuvenate the area naturally.

b. Vertical preperiosteal mid-face lift versus concentric malar lift

In 1992, Hamra [27] published his vertical elevation of the mid-face. The technique proposed a preperiosteal dissection, maintaining the origin of the muscles on the bone, associated with an arcus marginalis release. The elevation was created by an orbicularis flap, based in the lateral canthus, passed under the lateral raphe and sutured to the periosteum of the lateral orbital rim. A small skin excision at the mid-pupilla line line was possible.

The concentric malar lift [7] as published in 2004 is also a vertical technique, but its main vector is the mid-pupilla line compared to Hamra’s at the lateral canthus, and concentric malar lift dissection is subperiosteal compared to preperiosteal in Hamra’s technique.

In the concentric malar lift, thanks to the subperiosteal dissection plane, a complete release of the muscles from the bone is performed with freeing of the arcus marginalis, which does not interrupt septi, vessels and nerves converging toward the medial canthus. Because the concentric elevation is mostly on the mid-pupilla line, skin removal of nearly 20 mm at the mid-pupilla line on a previously non-operated case is achieved in most cases. Because of its concept, the concentric malar lift offers an improvement of the malar mound and in the skin tension in the whole malar area. Indeed, the concentric malar lift, with its subperiosteal dissection of the malar area, involves the only dissection plane that does not slip with time, as stated by Tessier [28], compared to the preperiosteal plane that relapses with time. Consequently, in the concentric malar lift, the reattachment of the malar tissues to a higher level is reliable.

c. Vertical subperiosteal mid-face lift versus concentric malar lift

In 2016, Botti [8] published a very interesting technique of vertical subperiosteal mid-face lift.

This publication adopts the idea of the vertical subperiosteal mid-face lift, first presented by the author in 2004 [1], but one of the main differences proposed by Botti is the complete opening of the orbicularis oculi muscle, following the skin opening from one canthus to the other.

This complete opening creates a direct connection between the eyelid margin and the anterior malar area. Consequently, in case of retraction over that large dissection area, which is not compartmentalized, a descent of the lower eyelid happens with no counteraction at the mid-pupilla line.

In the concentric malar lift [1], the muscular opening is limited at the lateral canthus, which is stabilized with the canthopexy and the muscular suspension. Even a major inflammatory retraction will not cause descent at the mid-pupillary level because of the conservation of the muscle insertion and only rarely and mildly involves the lateral canthus area. Massage and injections resolve the situation as previously explained.

The authors of the periorbital anchoring technique [8] outline that ‘the simple manipulation of palpebral fat… can in fact result in retractive scar formation and fusion of the orbital septum with the capsulopalpebral fascia posteriorly or with the orbicularis muscle and skin anteriorly.’ If this is true with a limited dissection, how can it be avoided with a large one?

The second difference is the degree of elevation of the mid-face. With 3 vertical fixations on the orbital rim, the periorbital anchoring technique permits removal up to 10–15 mm of skin excess. With this evolution of the concentric malar lift, this distance is increased because vertical fixation has been transformed into a ‘hammock’-like sling allowing en bloc elevation and the removal of 20–30-mm skin excess. This makes the concentric malar lift two times more efficient than the vertical anchoring technique.

In the author’s opinion, this review of competing techniques gives the advantage to the concentric malar lift, even if the learning curve is steep.

#### Analysis of Concentric Malar Lift Limitations and Complications

Besides the learning curve, one of the main limitations of the concentric malar lift is its limited effect on nasolabial volume.

If the patient’s face is round and if the skin excess in the nasolabial area is moderate, the direct elevation due to the first pass of suture, associated with skin tension, can give good improvement. But if the patient’s face is slim and the nasolabial fold has an overlying skin excess, even if 3 cm of skin is removed at the eyelid margin, the improvement at the nasolabial fold level remains inadequate. The addition of medio-jugal fat grafting may improve the result. In men, direct excision of skin from prominent nasolabial folds may be appropriate.

It is agreed that the main reason for the concentric malar lift not being adopted more widely, despite its positive results, is the number of key points to be taken into account for the surgeon and the learning curve. The learning process needs not only a clear understanding of the operative details and their logic, but also to include several visits to observe an experienced colleague first hand. Indeed, being an advanced and sophisticated technique, the concentric malar lift requires a traditional apprenticeship to acquire the skills to perform it.

The risk of chemosis (11%) is increased with the malar dissection and canthopexy. If chemosis is detected at the end of the operation, a lateral tarsorrhaphy can be performed. Patients need to be informed preoperatively of this possibility.

To reduce the risk of this complication, a preoperative visit to an ophthalmologist is recommended if the patient has not been seen by one in the recent past. A ‘clean bill of health’ from an ophthalmologist is required if the patient reports any preexisting ocular symptoms.

All patients follow a specific pre- and postoperative treatment protocol:

Two days before surgery, antiseptic and anti-inflammatory eye drops 3 times a day.

In the postoperative period, cleaning with saline and antibiotic eye drops is used for 3 days together with steroid and hyaluronic acid eye drops for 15 days.

In persistent chemosis, as previously explained, a preperiosteal injection of long acting steroid into the lateral part of the mid-cheek furrow is performed as an office procedure.

Transitory numbness of the infra-orbital territory is a specific complication of the technique (0.06%). It results from trauma to a branch of the infra-orbital nerve during surgery. If the nerve is not divided, it will recover spontaneously. While not visible, this complication can result in involuntary biting of the lip.

To prevent division of a branch, two precautions are recommended: keeping the dissector constantly in contact with the bone during the dissection and locating the infra-orbital nerve foramen with a thin hypodermic needle. If locating the foramen is difficult, the buccal mucosal incision should be used to ensure direct visualization of the nerve.

If these principles are observed, the risk of numbness is reduced and, if it does occur, should recover spontaneously.

Transitory weakness of the zygomatic branch of the facial nerve can occur in all mid-facial procedures involving malar elevation.

The zygomatic branch of the facial nerve is located superficially just below the inferior border of the malar. It is not dissection, but the suspension, which produces the problem. Unfortunately, any thread that is passed from deep to superficial to the skin, in order to elevate the tissues with a loop, can damage this zygomatic branch due to pressure caused by the elevation.

Consequently, when performing a concentric malar lift, threads must bite the periosteum and the deep fat but must not include more superficial muscles and nerves, avoiding a zygomatic branch palsy. In case of zygomatic palsy, an electromyography should be performed to follow the recovery.

### II/Reconstructive Discussion

The advantages of the mid-face lift for lower lid retraction are multiple with no scar from the harvested area, no difference in color or in texture.

Retraction of the lower eyelid has a range of causes.

It is well known that the most common complication after blepharoplasty is lower eyelid malposition (from scleral show to ectropion), with published rates of 5–30% [2–4].

For minor anterior lamella retraction, subcutaneous dissection of the lower lid must be at least half the distance between the eyelid margin and the mid-cheek junction. The malar elevation and this skin dissection will produce enough skin excess to achieve good elevation of the eyelid margin. The quantity of skin removed must take into account the quantity of skin needed to reposition the eyelid.

For a major anterior lamella retraction, no skin should be removed. If an extensive cutaneous deficit cannot be adequately compensated by elevation of the mid-face flap, a skin graft or a local flap is alternative procedures [6, 8].

For a posterior lamella retraction, an appropriately sized spacer graft harvested using hard palate/mucosal grafts is recommended, while for the medium lamella, either a thin temporal fascial graft or a hard palate graft is recommended [8].

If the canthopexy does not support the lower eyelid sufficiently, the well-known tarsal strip [29] technique with shortening of the lateral extremity of the lid is performed.

In cases of reconstructive malar lift, the risks are identical to when it is used cosmetically (chemosis, transitory numbness of the infra-orbital nerve). It must be noted that the risk of recurrence of retraction is increased in cases of reoperation for scarring.

### III/Performing the Concentric Malar Lift Safely

The key points to performing the concentric malar lift safely are:

1. Lateral muscle opening for access to the subperiosteal dissection must remain limited to avoid the risk of a complete lower eyelid descent in patients who scar badly. This incision must be located at the level of the lateral canthus where it will be possible to stabilize the tarsus and the posterior lamella with a canthopexy and the anterior lamella muscle with muscle elevation. No skin excision should be performed in the canthal area. Skin is only excised in the mid-pupilla line to avoid secondary canthal malposition.
2. Subperiosteal dissection reduces the risk of bleeding as much as possible.
  To limit bleeding the periosteum is injected with the solution already described containing a high concentration of epinephrine (0.5 mg per 300 ml of saline) with ropivacaine 7.5 mg in 20 ml and 20 mg/ml lidocaine with epinephrine 0.005 mg/ml.
  The subperiosteal dissection must be carefully performed, particularly at the inferior border.
3. Using 2 passes of a permanent Quill suture [15], the relocation of the mid-cheek volume is much better and malar mound much flatter. Some key elements for safety and efficacy of the two hammock suspensions are mandatory:
  it is performed just above the periosteum elevating deep tissues, without taking a bite of the muscles and nerves
  with a long hammock distance, the result is more efficient,
  one low pass elevates ‘en bloc’ the whole malar area and a second, higher pass, elevates the malar mound concentrically.
  the threads will not be palpable because they are placed on the bone.
4. A learning curve is normal, taking care to realize each step like described, because variations could be sources of complication.

## Conclusion

Since publication in 2004, the concentric malar lift has demonstrated its efficiency and reliability in both aesthetic rejuvenation and also reconstructive cases of lower eyelid retraction. The procedure has evolved, and the concentric malar lift is now a well-defined, safe and replicable technique.

## References

[1] Le Louarn C (2004). The concentric malar lift: malar and lower eyelid rejuvenation. Aesthet Plast Surg.

[2] Patipa M (2000). The evaluation and management of lower eyelid retraction following cosmetic surgery. Plast Reconstr Surg.

[3] Klatsky SN, Manson P, Goldwyn R, Cohen M (2001). Blepharoplasty. The unfavorable result in plastic surgery: avoidance and treatment.

[4] McCord CD, Boswell CB (2003). Hester TR Lateral canthal anchoring. Plast Reconstr Surg.

[5] Tonnard PL, Verpaele AM, Zeltzer AA (2013). Augmentation blepharoplasty: a review of 500 consecutive patients. Aesthet Surg J.

[6] Bartley GB (1996). The differential diagnosis and classification of eyelid retraction. Ophthalmology.

[7] Botti G, Pelle Ceravolo M (2012). Midface and neck aesthetic plastic surgery.

[8] Pascali M, Botti C, Cervelli V, Botti G (2017). Vertical midface lifting with periorbital anchoring in the management of lower eyelid retraction: a 10-year clinical retrospective study. Plast Reconstr Surg.

[9] Le Louarn C (1989). The malar musculo-fatty flap. Ann Chir Plast Esthet.

[10] Le Louarn C (1989). Surgical treatment of rings around the eyes. Ann Chir Plast Esthet.

[11] Le Louarn C (1992) The malar S.m.a.s. In: Hinderer UT (ed) Flap plastic surgery, vol II. pp 135–136

[12] Le Louarn C (1994). Incision cutanée dans le lifting. Lambeau de SMAS cervico malaire oblique et lifting malaire. Ann Chir Plast Esth.

[13] Le Louarn C, Buthiau D, Buis J (2006). Facial rejuvenation and concentric malar lift: the FACE RECURVE concept. Ann Chir Plast Esthet.

[14] Le Louarn C, Buthiau D, Buis J (2007). The face recurve concept: medical and surgical applications. Aesthet Plast Surg..

[15] Hamra ST (1998). The zygorbicular dissection in composite rhytidectomy: an ideal midface plane. Plast Reconstr Surg.

[16] Le Louarn C (2009). Midface region: functional anatomy, ageing process, indications and concentric malar lift. Ann Chir Plast Esthet.

[17] Le Louarn C (2015). Specificity of facelift surgery, including mid facelift, in case of facial palsy. Ann Chir Plast Esthet.

[18] de la Plaza R, de la Cruz L (2001). The sliding fat pad technique with use of the transconjunctival approach. Aesthet Surg J..

[19] Wong CH (2015). Mendelson B midcheek lift using facial soft-tissue spaces of the midcheek. Plast Reconstr Surg..

[20] Teo L, Woo YJ, Kim DK, Kim CY, Yoon JS (2017). Surgical outcomes of porcine acellular dermis graft in anophthalmic socket: comparison with oral mucosa graft. Korean J Ophthalmol.

[21] Shoukath S, Taylor GI, Mendelson BC, Corlett RJ, Shayan R, Tourani SS, Ashton MW (2017). The lymphatic anatomy of the lower eyelid and conjunctiva and correlation with postoperative chemosis and edema. Plast Reconstr Surg.

[22] Rohrich RJ, Ghavami A, Constantine FC, Unger J, Mojallal A (2014). Lift-and-fill 445 face lift: integrating the fat compartments. Plast Reconstr Surg.

[23] Jang MS, Kim HY, Dhong HJ, Chung SK, Hong SD, Cho HJ (2015). An analysis 448 of Asian midfacial fat thickness according to age group using 449 computed tomography. J Plast Reconstr Aesthet Surg.

[24] Gosain AK, Klein MH, Sudhakar PV, Prost RW (2005). A volumetric analysis of soft-tissue changes in the aging midface using high-resolution MRI: implications for facial rejuvenation. Plast Reconstr Surg.

[25] Ezure T, Hosoi J, Amano S, Tsuchiya T (2009). Sagging of the cheek is related to skin elasticity, fat mass and mimetic muscle function. Skin Res Technol.

[26] Owsley JQ, Roberts CL (2008). Some anatomical observations on midface aging and long-term results of surgical treatment. Plast Reconstr Surg.

[27] Hamra ST (1992). Composite rhytidectomy. Plast Reconstr Surg.

[28] Tessier P (1989). Subperiosteal face-lift. Ann Chir Plast Esthet.

[29] Anderson RL, Gordy DD (1979). The tarsal strip procedure. Arch Ophthalmol.

